# The Role of Astrocytic Calcium Signaling in the Aged Prefrontal Cortex

**DOI:** 10.3389/fncel.2018.00379

**Published:** 2018-11-05

**Authors:** Sónia Guerra-Gomes, João Filipe Viana, Diana Sofia Marques Nascimento, Joana Sofia Correia, Vanessa Morais Sardinha, Inês Caetano, Nuno Sousa, Luísa Pinto, João Filipe Oliveira

**Affiliations:** ^1^Life and Health Sciences Research Institute (ICVS), School of Medicine, University of Minho, Braga, Portugal; ^2^ICVS/3B’s – PT Government Associate Laboratory, Braga, Portugal; ^3^IPCA-EST-2Ai, Applied Artificial Intelligence Laboratory, Polytechnic Institute of Cávado and Ave, Campus of IPCA, Barcelos, Portugal

**Keywords:** aging, astrocyte, calcium signaling, IP_3_R2, prefrontal cortex, spatial recognition, dendritic morphology

## Abstract

Aging is a lifelong process characterized by cognitive decline putatively due to structural and functional changes of neural circuits of the brain. Neuron-glial signaling is a fundamental component of structure and function of circuits of the brain, and yet its possible role in aging remains elusive. Significantly, neuron-glial networks of the prefrontal cortex undergo age-related alterations that can affect cognitive function, and disruption of glial calcium signaling has been linked with cognitive performance. Motivated by these observations, we explored the possible role of glia in cognition during aging, considering a mouse model where astrocytes lacked IP_3_R2-dependent Ca^2+^ signaling. Contrarily to aged wild-type animals that showed significant impairment in a two-trial place recognition task, aged IP_3_R2 KO mice did not. Consideration of neuronal and astrocytic cell densities in the prefrontal cortex, revealed that aged IP_3_R2 KO mice present decreased densities of NeuN^+^ neurons and increased densities of S100β^+^ astrocytes. Moreover, aged IP_3_R2 KO mice display refined dendritic trees in this region. These findings suggest a novel role for astrocytes in the aged brain. Further evaluation of the neuron-glial interactions in the aged brain will disclose novel strategies to handle healthy cognitive aging in humans.

## Introduction

Aging associates cognitive decline involving decrease of attention, working memory capacity, inhibitory control, and speed processing (Rajah and D’Esposito, 2005; Grady Cheryl, 2008; Bizon et al., 2012). Several factors may contribute to the installation of selective cognitive impairments. Recent literature suggests that changes in cellular morphology, signaling, and gene expression may disrupt the network dynamics of aged prefrontal circuits, leading to cognitive dysfunction (Burke and Barnes, 2006). While the available literature agrees that changes in neuronal structure are tightly linked to cognitive alterations, there is still controversy in the field regarding the type of morphological changes that occur (Konsolaki and Skaliora, 2015). This controversy may be partially justified by the biological variability of subjects and brain populations across the long-lasting aging process, as well as the variety of experimental approaches used for the analysis (Engle and Barnes, 2012; Lemaitre et al., 2012; Jagust, 2013). Moreover, recent reports highlight age-dependent changes in glial signaling that could modulate network computation with impact in cognitive performance (Soreq et al., 2017; Boisvert et al., 2018). Being the most abundant type of glial cell in the central nervous system, astrocytes can sense, process and respond to incoming signals, modulating the extracellular milieu and transmission of neural signals (Araque et al., 2014). Significantly, astrocytes seem crucially involved in structural and functional integrity of neural circuits of the prefrontal cortex (PFC) – a brain area chiefly involved in cognition – related tasks that are affected by aging. Ablation of PFC astrocytes for example, results in detrimental effects in different behavior domains. Specifically, astrocyte ablation in this region induces an anxious and depressive phenotype (Banasr and Duman, 2008) and is responsible for impairments in spatial working memory, attention, and behavioral flexibility (Lima et al., 2014). Although astrocytic features during both development and adulthood are well studied, its implications for the aging brain are still poorly understood. Together with microglia, astrocytes may exert beneficial or detrimental influence onto neuronal circuits and therefore impact the aging process (Lynch et al., 2010). Recently, it was shown that neuron-glia signaling remains conserved during brain aging and that astrocytic intracellular calcium (Ca^2+^) elevations are maintained during lifetime (Gómez-Gonzalo et al., 2017). This is important since astrocytes respond to synaptic activity by increasing their intracellular Ca^2+^ levels within a spatio-temporal scale (Araque et al., 2014; Volterra et al., 2014). This physiological hallmark of the astrocytic response has a functional impact in several brain regions, specifically in synapses, circuits, and behavior (Oliveira et al., 2015; Guerra-Gomes et al., 2017). Astrocytic Ca^2+^ signaling (and Ca^2+^-dependent pathways) is intimately involved in the control of synaptic transmission and plasticity in brain regions responsible for learning and memory processing (Henneberger et al., 2010; Tanaka et al., 2013), however, the direct cognitive consequences are still poorly understood (Guerra-Gomes et al., 2017). Ca^2+^ elevations in astrocytes range from global Ca^2+^ elevations in the soma to focal Ca^2+^ events at thinner processes (Kanemaru et al., 2014; Srinivasan et al., 2015; Agarwal et al., 2017; Bindocci et al., 2017; Stobart et al., 2018; Yu et al., 2018). A predominant component of this signaling is by the Ca^2+^ release from the endoplasmic reticulum via IP_3_ receptor channels (IP_3_Rs) (Volterra et al., 2014; Bazargani and Attwell, 2016). With this regard, immunohistochemistry and transcriptomic analysis revealed that type 2 IP_3_Rs (IP_3_R2) are mainly expressed in astrocytes (Sharp et al., 1999; Hertle and Yeckel, 2007; Takata et al., 2011; Zhang et al., 2014; Li et al., 2015). They are the major source of ER-dependent global astrocyte Ca^2+^, and mice lacking IP_3_R2 display “silent” astrocytes with minimal Ca^2+^ elevations in the soma and main processes (Petravicz et al., 2008; Takata et al., 2011; Navarrete et al., 2012). Leveraging on these findings, we study cognitive performance of IP_3_R2 KO mice to explore on the importance of IP_3_R2-dependent Ca^2+^ signaling in astrocytes during aging. Our results show that aged IP_3_R2 KO mice display a preserved cognitive performance in a PFC dependent task, along with neuronal dendritic refinement and reduced neuron-to-astrocyte ratios in this brain region.

## Materials and Methods

### Animals

All experimental procedures were conducted in accordance with the guidelines described in Directive 2010/63/EU and were approved by the local ethical committee (SECVS 075/2015) and Portugal national authority for animal experimentation (DGAV 17469/2012). IP_3_R2 KO mice were kindly supplied by Prof. Alfonso Araque (University of Minnesota, United States) (Navarrete et al., 2012), under agreement with Prof. Ju Chen (University of San Diego, United States) (Li et al., 2005). Mice were backcrossed to C57BL/6J for at least five generations in our lab. IP_3_R2 KO and their respective littermate wild-type (WT) controls were obtained by mating IP_3_R2^+/−^ mice. Male WT and IP_3_R2 KO mice of 2- to 3-month-old (adults) and 18- to 19-month-old (aged) were used for the experiments. They received an individual tag, which remained unaltered throughout the experiment and allowed to perform the behavioral and histological evaluation in a blind manner. All mice had *ad libitum* access to food and water in their home cages and lights were maintained on a 12 h light/dark cycle (lights on 8:00 A.M. to 8:00 P.M.) at 22 ± 1°C, 55% humidity.

### Two-Trial Place Recognition Task

The Y-maze two-trial place recognition (2TPR) task evaluates spatial recognition memory, a form of episodic-like memory, by taking advantage of the innate propensity of rodents to explore novel environments, as previously described (Sardinha et al., 2017). The maze was composed by three equal arms (33.2 L × 7 W × 15 cm H), made of white Plexiglas. To increase spatial recognition and navigation, the end of each arm contained a visual cue. Each mouse was initially placed at the end of the Start (S) arm and it was allowed to freely explore this arm and an additional arm (Familiar arm, F) for 5 min. In the second trial, a divider was removed allowing the exploration of a novel (N) arm, and each mouse was allowed to explore the three arms for 2 min for memory retrieval. The test was performed in dim light conditions and the maze was cleaned with 10% ethanol between subjects. All trials were acquired and analyzed using a video-tracking system (Videotrack; Viewpoint) and the EthoVision XT 12 software (Noldus, Netherlands). Data was expressed as a discrimination index (D.I.) of time and distance, calculated for the distal third of each arm using the following equation (1):

$$\text{D}.\text{I}.\text{=}\frac{Novel-\frac{Start+Familar}{2}}{Novel+\frac{Start+Familar}{2}}\;(1)$$

A positive D.I. indicates preference for the novel arm, meaning that mice retained a memory of the arms previously explored (start and familiar), and therefore display a better spatial recognition performance.

### Tissue Processing and Immunohistochemical Analysis

Mice were anesthetized with a mixture of ketamine (75 mg/kg, i.p.; Imalgene 1000, Merial, United States) and medetomidine (1 mg/kg, i.p.; Dorbene Vet, Pfizer, United States), and transcardially perfused with 0.9% saline. Brains were carefully removed, fixed overnight with 4% paraformaldehyde and immunofluorescence experiments were performed in cryostat coronal brain sections (20 μm thick). Sections were incubated overnight with the primary antibodies: rabbit anti-S100β (1:200, DakoCytomation; AB_2315306) and rabbit anti-NeuN (1:100, Cell Signaling Technology; AB_2651140). In the next day, incubation with the secondary antibody Alexa Fluor^®^ 594 donkey anti-rabbit (1:1000, Thermo Fisher Scientific; AB_2556543) was carried out. Images were acquired in an Olympus Fluoview FV1000 confocal microscope (Olympus, Hamburg, Germany), and the number of S100β^+^ and NeuN^+^ cells was calculated using the ImageJ plugin – “Cell counter^1^.”

### Three Dimensional-Reconstruction of mPFC Layer V Pyramidal Neurons

Three dimensional (3D) dendritic morphology was assessed in Golgi-Cox stained material as previously described (Lima et al., 2014). mPFC layer V pyramidal neurons were analyzed for the following dendritic features: length, arborization, Sholl analysis and spine number and classification. Briefly, at least five neurons were analyzed for each animal by using a motorized microscope controlled by the Neurolucida software (MBF Bioscience, United States) under 100× magnification. Dendritic spine densities were assessed in randomly selected dendritic segments of 30 μm, in the proximal and distal portions of the apical dendrite and in the proximal portion of the basal dendrite. Moreover, spines were classified into four categories: thin, mushroom, thick, and ramified. The extraction of data for both reconstructed neurons and spines was performed by using NeuroExplorer software (MBF Bioscience, United States).

### Statistical Analysis

Statistical analysis was performed using the GraphPad Prism 6.01 (GraphPad Software Inc., United States). All data analyzed passed the D’Agostino and Pearson normality test for Gaussian distributions. Two-way analysis of variance (ANOVA) with Sidak *post hoc* test was applied to analyze the performance in the Y-maze 2TPR, cell densities, neuronal length, and endings, considering either factor: genotype or age. Two-way ANOVA with Tukey’s multiple comparisons test was used to analyze Sholl analysis data for neuronal 3D reconstructions. Data are presented throughout the manuscript as mean ± SEM (Standard Error of the Mean) and results were considered significant for *p* < 0.05.

## Results

### Lack of IP_3_R2-Dependent Astrocytic Calcium Prevents Age-Related Cognitive Decline

**FIGURE 1:**
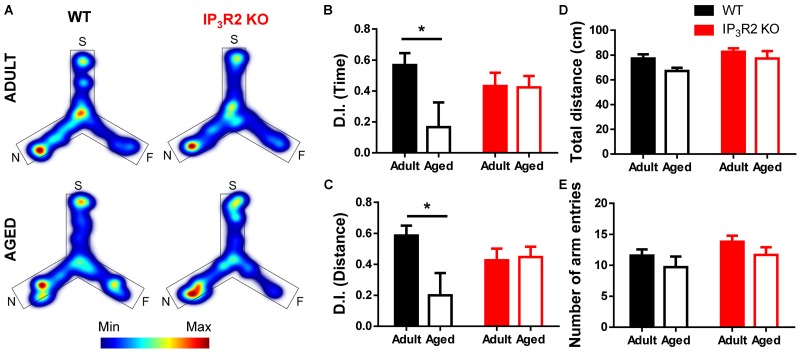
Lack of IP_3_R2-dependent astrocytic calcium prevents age-related cognitive decline in a PFC-dependent task. **(A)** Representative heatmaps of cumulative time exploration of start (S), familiar (F), and novel (N) arms of the Y-maze for WT (left, black) and IP_3_R2 KO (right, red) mice (warm colors, more time; cold colors, less time); Discrimination index (D.I.) for time **(B)** and distance **(C)**, representative of the spatial preference for the novel arm in the retrieval trial. **(D)** Total distance traveled in the maze; **(E)** Total number of entries in the three arms. Adult mice are represented in filled bars; aged mice are represented in unfilled bars. *n* = 9–13 per group, two-way ANOVA, Sidak’s multiple comparisons test; data plotted as mean ± SEM. ^∗^*p* < 0.05.

We tested the performance of both WT and IP_3_R2 KO mice (adult and aged) in a PFC-dependent task. We performed the 2TPR task which evaluates spatial recognition memory in mice, based on their natural drive to explore novelty (Figure 1). This test has the advantage of being physically less demanding, and therefore more suitable to assess cognition in aged mice (Pistell et al., 2012; Dolgin, 2013). Our results show that aged WT mice display a deficit in recognition memory, since they retain less memory of the familiar arms and fail to discriminate the novel arm when compared to their adult WT littermates (Figures 1A,B; Sidak *post hoc* test; *p* < 0.05). Surprisingly, this deficit in spatial recognition memory is not observed in aged IP_3_R2 KO mice, as those animals explored the novel arm longer, similarly to their adult counterparts (Figures 1A,B). In accordance, aged WT mice walk less distance in the novel area than their adult WT littermates (Figures 1A,C; Sidak *post hoc* test; *p* < 0.05), a deficit not observed in aged IP_3_R2 KO mice. The Sidak *post hoc* comparison between genotypes discarded any significant difference for both measures (time or distance), excluding an effect of IP_3_R2 KO in the performance between adult or aged mice in this experimental setup. Importantly, tested mice equally explored the maze during the task, as given by their total distance traveled (Figure 1D) and number of arm entries (Figure 1E), regardless of their genotype or age, hence excluding any age-related loss of their natural exploratory drive.

Together, these results point out an unpredicted maintenance of PFC-dependent cognitive performance in aged mice that lack IP_3_-dependent astrocytic Ca^2+^ signaling.

### Aging Leads to a Decrease in Neuronal but Not Glial Densities in IP_3_R2 KO Mice

**FIGURE 2:**
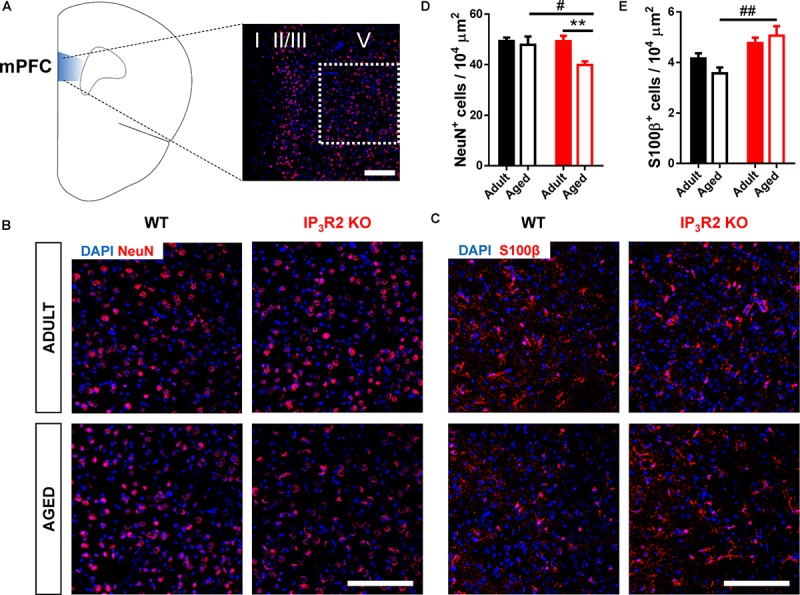
Aged IP_3_R2 KO mice display decreased NeuN^+^ neuron densities and increased S100β^+^ astrocyte densities. **(A)** Scheme of the prelimbic subregion of the medial prefrontal cortex (mPFC, blue) and respective layers I, II/III, and V. mPFC layer V (white dash line) corresponds to the area considered for cell counting. **(B)** Representative mPFC layer V confocal images of DAPI/NeuN labeling for WT and IP_3_R2 KO mice (adult and aged). **(C)** Representative mPFC layer V confocal images of DAPI/S100β labeling for WT and IP_3_R2 KO mice (adult and aged); **(A–C)** scale bars = 200 μm. **(D,E)** Densities of NeuN^+^ **(D)** and S100β^+^ **(E)** cells in mPFC layer V of WT and IP_3_R2 KO mice (adult and aged; *n* = 8–15 images per group; two-way ANOVA, Sidak’s multiple comparisons test). WT (adults and aged) mice are represented in filled/unfilled black bars, whilst IP_3_R2 KO (adults and aged) are represented in filled/unfilled red bars. Data plotted as mean ± SEM. ^#^*p* < 0.05 and ^##^*p* < 0.01 between genotypes and ^∗∗^*p* < 0.01 between ages.

To correlate the observed behavioral phenotype with possible alterations in the populations of neurons and astrocytes, we estimated the density of cells, respectively, stained for NeuN^+^ and S100β^+^ markers. NeuN is a well-known marker for post-mitotic neurons (Mullen et al., 1992), whereas S100β is a recognized marker for astrocytes (Wang and Bordey, 2008). We assessed these cellular densities in layer V of the medial PFC (Figure 2A), which is the main prefrontal output to other cortical and subcortical regions involved in cognitive behavior (Opris and Casanova, 2014; Naka and Adesnik, 2016), and receives a main hippocampal input involved in episodic memory with a strong spatial component (Hoover and Vertes, 2007; Barker et al., 2017) required for the performance in the 2TPR task. S100β^+^ cells were previously shown to colocalize with IP_3_R2 in the rodent brain (Sharp et al., 1999; Takata et al., 2011; Li et al., 2015). Our analysis indicated an effect of aging on NeuN^+^ cell density (Figures 2B,D; age: *F*_1,33_ = 6.192, *p* = 0.018), which may be accounted by a pronounced decrease of NeuN^+^ cells observed in aged IP_3_R2 KO mice when compared with their aged WT littermates (Figure 2D; Sidak *post hoc* test; *p* < 0.05). Moreover, this reduction in the number of neuronal cells in IP_3_R2 KO mice is also significantly different from their adult IP_3_R2 KO mice (Sidak *post hoc* test; *p* < 0.01), that maintain similar cellular densities to WT mice.

On the contrary, we found an overall increase of S100β^+^ astrocytes in IP_3_R2 KO mice (Figures 2C,E; genotype: *F*_1,40_ = 16.19, *p* < 0.05). Contrarily to what we observed for neurons, we found an increase in S100β^+^ cells in aged IP_3_R2 KO when compared to aged WT mice (Sidak *post hoc* test; *p* < 0.01). Altogether, these results suggest that lack of age-related cognitive impairment in the absence of IP_3_R2-mediated Ca^2+^ signaling in astrocytes, correlates with a different neuron-to-glia ratio in the PFC which could ultimately result either in different neuron-glial circuits, or functional neuron-glia interactions or both.

### Aging Leads to a Dendritic Refinement of mPFC Layer V Pyramidal Neurons in IP_3_R2 KO Mice

**FIGURE 3:**
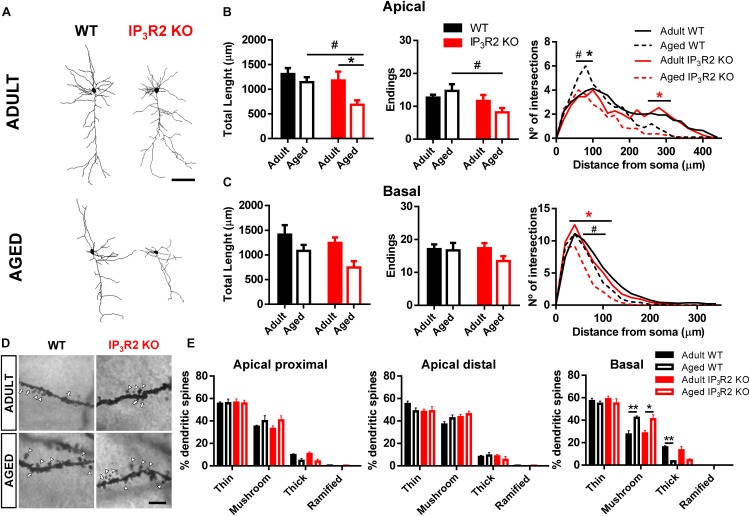
Aging leads to a dendritic refinement of mPFC layer V pyramidal neurons in IP_3_R2 KO mice. **(A)** Representative 3D reconstructions of layer V mPFC neurons from WT and IP_3_R2 KO mice (adults or aged; scale bar = 100 μm). **(B,C)** Morphological parameters of apical **(B)** and basal **(C)** dendrites by assessing total dendritic length, number of endings and Sholl intersections (*n* = 10–14 neurons per group; two-way ANOVA, Sidak’s multiple comparisons test). **(D)** Representative images of basal dendritic segments of each experimental group; scale bar = 5 μm; arrowheads, mushroom-type; arrow, thick-type. **(E)** Percentage of each spine type at proximal and distal segments of apical dendrites, and basal dendrites (*n* = 6–14 neurons per group; two-way ANOVA, Tukey’s multiple comparisons test). WT (adults and aged) mice are represented either by filled/unfilled black bars or black line/dashed line, while IP_3_R2 KO (adults and aged) are represented either by filled/unfilled red bars or red line/dashed line. Data plotted as mean ± SEM. ^∗^Denotes the effect of age; ^#^denotes the effect of genotype. Sholl analysis: black ^∗^, refers to difference between WT; red ^∗^, refers to difference between IP_3_R2 KO; #, ^∗^*p* < 0.05; ^∗∗^*p* < 0.01.

An important component underpinning neuron-glial interaction, is the neuropil morphology to the extent that it may define structural and functional constraints of such interaction (Volterra et al., 2014; De Pittà et al., 2016). With this regard, we considered dendrite morphology for WT vs. IP_3_R2 KO and adult vs. aged mice (Figure 3A). Significantly, both age and lack of IP_3_R2 lead to a decrease in apical dendrite length of 3D-reconstructed Golgi-impregnated neurons (Figure 3B; age: *F*_1,42_ = 6.340, *p* = 0.016; genotype: *F*_1,42_ = 5.168, *p* = 0.028), which was accompanied by a reduction of apical endings in mice that lack IP_3_R2 (*F*_1,42_ = 6.259, *p* = 0.016). In line with neuronal density data, these effects were caused by the marked reductions of apical dendrites of layer V pyramidal neurons observed in aged IP_3_R2 KO mice. Specifically, aged IP_3_R2 KO pyramidal neurons display a shorter and less ramified morphology, as compared with aged WT mice (Sidak *post hoc* test; *p* < 0.05). Furthermore, when compared with their adult IP_3_R2 KO littermates, aged IP_3_R2 KO mice also revealed significantly reduced apical dendritic length (Sidak *post hoc* test; *p* < 0.05). These alterations are detailed by the Sholl analysis data, which revealed an overall effect of radius, genotype and an interaction between these two factors (radius: *F*_22,924_ = 41.86, *p* < 0.0001; genotype: *F*_3,42_ = 4.089, *p* = 0.012; interaction: *F*_66,924_ = 1.821, *p* = 0.0001). *Post hoc* analysis showed that aged IP_3_R2 KO mice display fewer intersections, as compared with aged WT and their adult genotype matches (Tukey *post hoc* test; *p* < 0.05).

The analysis of basal dendritic morphology in neurons from the same layer V revealed that although they seem to be also reduced in aged IP_3_R2 KO mice, this is only significant in the overall comparison by age and in the detailed Sholl analysis. Specifically, aging seems to lead to dendritic shortening (Figure 3C; *F*_1,42_ = 7.230, *p* = 0.010). Moreover, the Sholl analysis revealed a significant effect of radius, genotype and an interaction between factors (radius: *F*_17,714_ = 160.4, *p* < 0.0001; genotype: *F*_3,42_ = 3.532, *p* = 0.023; interaction: *F*_51,714_ = 2.055, *p* < 0.0001). Additional analysis showed a decreased number of intersections around 40–100 μm from the soma in aged IP_3_R2 KO, as compared with aged WT and adult IP_3_R2 KO mice (Tukey *post hoc* test; *p* < 0.05).

Since dendritic remodeling frequently has consequences for spine stability, we further analyzed the implication of IP_3_R2-dependent signaling to spine integrity upon aging. For that, we performed dendritic spine categorization at the apical proximal, apical distal, and basal segments of these neurons (Figures 3D,E). We observed the typical distribution of spines in the three dendritic segments, being the thin and mushroom types much more abundant than thick or ramified types: apical proximal (*F*_3,99_ = 386.1, *p* < 0.0001); apical distal (*F*_3,99_ = 312.6, *p* < 0.0001); basal (*F*_3,99_ = 250.9, *p* < 0.0001). Besides this expected spine distribution, aging led to an increase in the mushroom type specifically in the basal dendrites in mice of both genotypes, which was accompanied by a decrease in the densities of thick spines (Tukey *post hoc* test; *p* < 0.05). This observation was also described previously (Burke and Barnes, 2006) and was independent of the lack of IP_3_R2.

## Discussion

In the present study we applied behavioral and morphological approaches to untangle the influence of IP_3_R2-dependent astrocytic Ca^2+^ in the aged PFC. We found memory impairment in aged WT mice, which is in accordance with several evidences pointing to a cognitive decline during aging (Rajah and D’Esposito, 2005; Grady Cheryl, 2008; Weber et al., 2015). Surprisingly, aged mice that lack astrocyte Ca^2+^ elevations (IP_3_R2 KO) perform similarly to WT mice in the same PFC-dependent task, suggesting that astrocytes are involved in the age-related cognitive decline.

Regarding the behavioral observations, the cognitive conservation of aged IP_3_R2 KO mice is in line with previous studies using the same mouse model that show a putative role for IP_3_R2-dependent Ca^2+^ signaling in neuroprotection and behavior conservation after brain damage (Li et al., 2015; Rakers and Petzold, 2017). More importantly, a recent study shows that the deletion of IP_3_R2 in a model of Alzheimer’s disease (typically associated with cognitive decline) leads to the retention of spatial learning and memory (Reichenbach et al., 2018). This suggests that astrocyte activity mediated by reticular Ca^2+^ elevations may influence the network structure and function along the aging process. Indeed, Boisvert et al. (2018) demonstrated that in spite of maintaining homeostatic and neurotransmission-regulating genes, aged astrocytes in the cortex partially resemble reactive astrocytes creating an environment permissive to synapse elimination and neuronal damage, possibly contributing to aging-associated cognitive decline.

The available literature appears to agree that normal aging does not lead to major neuronal loss in most cortical regions (Burke and Barnes, 2006; Bishop et al., 2010). Besides, the neuron-glia signaling seems to remain conserved during lifespan (Gómez-Gonzalo et al., 2017). Our data are in agreement with these evidences, since both neuronal (NeuN^+^) and astrocyte (S100β^+^) densities remain unchanged in aged WT mice. Curiously, aged IP_3_R2 KO mice – that retain the cognitive ability in a PFC-dependent task – display a reduction in neuronal densities, while astrocyte densities display an inverse tendency in the same region, when compared to the observations of us and others at the adult stage (Li et al., 2015). These findings may be linked since overexpression of astrocyte markers in aged mice has been associated with its detrimental effects to neuronal networks in several cortical areas, namely through astrogliosis (Lynch et al., 2010; Orre et al., 2014; Rodríguez et al., 2014; Rogers et al., 2017; Boisvert et al., 2018).

While healthy aging is linked with mild or subtle morphological changes, pathological aging is rather linked to drastic reductions of neuronal structures (Hof and Morrison, 2004; Dickstein et al., 2007; Konsolaki and Skaliora, 2015). In this work, aged IP_3_R2 KO mice display a marked reduction of dendritic tree complexity, namely in apical dendrites, which appears to be related with the retention of their cognitive abilities as they age. Although our data does not provide a causal link between structural changes (dendritic simplification and different cell densities) and “silenced” age-related astrocyte signals, we believe that the preserved cognitive performance is rather a result of both. These neuronal alterations may result from changes in activation and signaling of a larger number of astrocytes (similarly to a mild astrogliosis), additional glial involvement (i.e., microglia, that play an extensive role in brain aging) (Lynch et al., 2010; Lalo et al., 2014; Das and Svendsen, 2015; Verkhratsky et al., 2016), and homeostatic regulatory mechanisms at the neuron network level (Wefelmeyer et al., 2016). It is noteworthy that our data indicate a great degree of spine stability in aged mice, namely at apical dendrites. Spine distribution and number in the PFC is tightly related with cognitive performance and evolves along the aging process (Burke and Barnes, 2006; Holtmaat and Svoboda, 2009; Bloss et al., 2011). In basal dendrites, aging triggers a shift to the mushroom type, which refers to a need for synaptic stability and complexity in aged layer V synapses. Nevertheless, this effect was visible in tissue of both WT and IP_3_R2 KO mice, which suggests that spine remodeling may not play a relevant role in the observed cognitive maintenance.

## Conclusion

In conclusion, the present study demonstrates that silencing the main source of global astrocytic Ca^2+^ leads to a conservation of the cognitive ability along aging. This cognitive resilience appears to be a product of gross neuronal structure refinement, together with an addition of astrocytes to the network. Whether other cell types and/or circuits might be involved in this cognitive maintenance is a matter that should be addressed in the future. Either way, disclosing further the astrocyte roles in the aging brain should be pivotal to provide alternative solutions to prevent or treat cognitive decline in humans.

## Author Contributions

SG-G, JV, DSMN, JC, VS, and IC designed, performed, and analyzed the experiments; made the figures; and wrote the manuscript. NS, LP, and JO supervised the study, wrote the manuscript, and secured funding.

## Conflict of Interest Statement

The authors declare that the research was conducted in the absence of any commercial or financial relationships that could be construed as a potential conflict of interest.

## Footnotes

1 http://fiji.sc/Fiji

## References

[B1] Agarwal A. Wu P.-H. Hughes E. G. Fukaya M. Tischfield M. A. Langseth A. J. (2017). Transient opening of the mitochondrial permeability transition pore induces microdomain calcium transients in astrocyte processes. *Neuron* 93 587.e7–605.e7. 10.1016/j.neuron.2016.12.034 28132831 PMC5308886

[B2] Araque A. Carmignoto G. Haydon P. G. Oliet S. H. R. Robitaille R. Volterra A. (2014). Gliotransmitters travel in time and space. *Neuron* 81 728–739. 10.1016/j.neuron.2014.02.007 24559669 PMC4107238

[B3] Banasr M. Duman R. S. (2008). Glial loss in the prefrontal cortex is sufficient to induce depressive-like behaviors. *Biol. Psychiatry* 64 863–870. 10.1016/j.biopsych.2008.06.008 18639237 PMC2709733

[B4] Barker G. R. I. Banks P. J. Scott H. Ralph G. S. Mitrophanous K. A. Wong L.-F. (2017). Separate elements of episodic memory subserved by distinct hippocampal–prefrontal connections. *Nat. Neurosci.* 20 242–250. 10.1038/nn.4472 28067902

[B5] Bazargani N. Attwell D. (2016). Astrocyte calcium signaling: the third wave. *Nat. Neurosci.* 19 182–189. 10.1038/nn.4201 26814587

[B6] Bindocci E. Savtchouk I. Liaudet N. Becker D. Carriero G. Volterra A. (2017). Three-dimensional Ca2 + imaging advances understanding of astrocyte biology. *Science* 356:eaai8185. 10.1126/science.aai8185 28522470

[B7] Bishop N. A. Lu T. Yankner B. A. (2010). Neural mechanisms of ageing and cognitive decline. *Nature* 464 529–535. 10.1038/nature08983 20336135 PMC2927852

[B8] Bizon J. L. P. D. Foster T. C. Alexander G. E. Glisky E. L. (2012). Characterizing cognitive aging of working memory and executive function in animal models. *Front. Aging Neurosci.* 4:19 10.3389/fnagi.2012.00019 PMC3439637 22988438

[B9] Bloss E. B. Janssen W. G. Ohm D. T. Yuk F. J. Wadsworth S. Saardi K. M. (2011). Evidence for reduced experience-dependent dendritic spine plasticity in the aging prefrontal cortex. *J. Neurosci.* 31 7831–7839. 10.1523/JNEUROSCI.0839-11.2011 21613496 PMC3398699

[B10] Boisvert M. M. Erikson G. A. Shokhirev M. N. Allen N. J. (2018). The aging astrocyte transcriptome from multiple regions of the mouse brain. *Cell Rep.* 22 269–285. 10.1016/j.celrep.2017.12.039 29298427 PMC5783200

[B11] Burke S. N. Barnes C. A. (2006). Neural plasticity in the ageing brain. *Nat. Rev. Neurosci.* 7 30–40. 10.1038/nrn1809 16371948

[B12] Das M. M. Svendsen C. N. (2015). Astrocytes show reduced support of motor neurons with aging that is accelerated in a rodent model of ALS. *Neurobiol. Aging* 36 1130–1139. 10.1016/j.neurobiolaging.2014.09.020 25443290

[B13] De Pittà M. Brunel N. Volterra A. (2016). Astrocytes: orchestrating synaptic plasticity? *Neuroscience* 323 43–61. 10.1016/j.neuroscience.2015.04.001 25862587

[B14] Dickstein D. L. Kabaso D. Rocher A. B. Luebke J. I. Wearne S. L. Hof P. R. (2007). Changes in the structural complexity of the aged brain. *Aging Cell* 6 275–284. 10.1111/j.1474-9726.2007.00289.x 17465981 PMC2441530

[B15] Dolgin E. (2013). Old mice require new experimental tricks to study aging process. *Nat. Med.* 19 518–519. 10.1038/nm0513-518 23652091

[B16] Engle J. R. Barnes C. A. (2012). Characterizing cognitive aging of associative memory in animal models. *Front. Aging Neurosci.* 4:10 10.3389/fnagi.2012.00010 PMC3439635 22988435

[B17] Gómez-Gonzalo M. Martin-Fernandez M. Martínez-Murillo R. Mederos S. Hernández-Vivanco A. Jamison S. (2017). Neuron–astrocyte signaling is preserved in the aging brain. *Glia* 65 569–580. 10.1002/glia.23112 28130845 PMC5314210

[B18] Grady Cheryl L. (2008). Cognitive neuroscience of aging. *Ann. N. Y. Acad. Sci.* 1124 127–144. 10.1196/annals.1440.009 18400928

[B19] Guerra-Gomes S. Sousa N. Pinto L. Oliveira J. F. (2017). Functional roles of astrocyte calcium elevations: from synapses to behavior. *Front. Cell. Neurosci.* 11:427. 10.3389/fncel.2017.00427 29386997 PMC5776095

[B20] Henneberger C. Papouin T. Oliet S. H. R. Rusakov D. A. (2010). Long-term potentiation depends on release of D-serine from astrocytes. *Nature* 463 232–236. 10.1038/nature08673 20075918 PMC2807667

[B21] Hertle D. N. Yeckel M. F. (2007). Distribution of inositol-1,4,5-trisphosphate receptor isotypes and ryanodine receptor isotypes during maturation of the rat hippocampus. *Neuroscience* 150 625–638. 10.1016/j.neuroscience.2007.09.058 17981403 PMC2238340

[B22] Hof P. R. Morrison J. H. (2004). The aging brain: morphomolecular senescence of cortical circuits. *Trends Neurosci.* 27 607–613. 10.1016/j.tins.2004.07.013 15374672

[B23] Holtmaat A. Svoboda K. (2009). Experience-dependent structural synaptic plasticity in the mammalian brain. *Nat. Rev. Neurosci.* 10 647–658. 10.1038/nrn2699 19693029

[B24] Hoover W. B. Vertes R. P. (2007). Anatomical analysis of afferent projections to the medial prefrontal cortex in the rat. *Brain Struct. Funct.* 212 149–179. 10.1007/s00429-007-0150-4 17717690

[B25] Jagust W. (2013). Vulnerable neural systems and the borderland of brain aging and neurodegeneration. *Neuron* 77 219–234. 10.1016/j.neuron.2013.01.002 23352159 PMC3558930

[B26] Kanemaru K. Sekiya H. Xu M. Satoh K. Kitajima N. Yoshida K. (2014). In vivo visualization of subtle, transient, and local activity of astrocytes using an ultrasensitive Ca(2+) indicator. *Cell Rep.* 8 311–318. 10.1016/j.celrep.2014.05.056 24981861

[B27] Konsolaki E. Skaliora I. (2015). Premature aging phenotype in mice lacking high-affinity nicotinic receptors: region-specific changes in layer v pyramidal cell morphology. *Cereb. Cortex* 25 2138–2148. 10.1093/cercor/bhu019 24554727

[B28] Lalo U. Rasooli-Nejad S. Pankratov Y. (2014). Exocytosis of gliotransmitters from cortical astrocytes: implications for synaptic plasticity and aging. *Biochem. Soc. Trans.* 42 1275–1281. 10.1042/BST20140163 25233403

[B29] Lemaitre H. Goldman A. L. Sambataro F. Verchinski B. A. Meyer-Lindenberg A. Weinberger D. R. (2012). Normal age-related brain morphometric changes: nonuniformity across cortical thickness, surface area and gray matter volume? *Neurobiol. Aging* 33 617.e1–617.e9. 10.1016/j.neurobiolaging.2010.07.013 20739099 PMC3026893

[B30] Li H. Xie Y. Zhang N. Yu Y. Zhang Q. Ding S. (2015). Disruption of IP3R2-mediated Ca2 + signaling pathway in astrocytes ameliorates neuronal death and brain damage while reducing behavioral deficits after focal ischemic stroke. *Cell Calcium* 58 565–576. 10.1016/j.ceca.2015.09.004 26433454 PMC4658295

[B31] Li X. Zima A. V. Sheikh F. Blatter L. A. Chen J. (2005). Endothelin-1–induced Arrhythmogenic Ca2 + signaling is abolished in atrial myocytes of inositol-1,4,5-Trisphosphate(IP3)–receptor type 2–deficient mice. *Circ. Res.* 96 1274–1281. 10.1161/01.RES.0000172556.05576.4c 15933266

[B32] Lima A. Sardinha V. M. Oliveira A. F. Reis M. Mota C. Silva M. A. (2014). Astrocyte pathology in the prefrontal cortex impairs the cognitive function of rats. *Mol. Psychiatry* 19 834–841. 10.1038/mp.2013.182 24419043

[B33] Lynch A. M. Murphy K. J. Deighan B. F. O’Reilly J.-A. Gun’ko Y. K. Cowley T. R. (2010). The impact of glial activation in the aging brain. *Aging Dis.* 1 262–278. 22396865 PMC3295033

[B34] Mullen R. J. Buck C. R. Smith A. M. (1992). NeuN, a neuronal specific nuclear protein in vertebrates. *Development* 116 201–211. 1483388 10.1242/dev.116.1.201

[B35] Naka A. Adesnik H. (2016). Inhibitory circuits in cortical layer 5. *Front. Neural Circ.* 10:35. 10.3389/fncir.2016.00035 27199675 PMC4859073

[B36] Navarrete M. Perea G. de Sevilla D. F. Gómez-Gonzalo M. Núñez A. Martín E. D. (2012). Astrocytes mediate in vivo cholinergic-induced synaptic plasticity. *PLoS Biol.* 10:e1001259. 10.1371/journal.pbio.1001259 22347811 PMC3279365

[B37] Oliveira J. F. Sardinha V. M. Guerra-Gomes S. Araque A. Sousa N. (2015). Do stars govern our actions? Astrocyte involvement in rodent behavior. *Trends Neurosci.* 38 535–549. 10.1016/j.tins.2015.07.006 26316036

[B38] Opris I. Casanova M. F. (2014). Prefrontal cortical minicolumn: from executive control to disrupted cognitive processing. *Brain* 137 1863–1875. 10.1093/brain/awt359 24531625 PMC4065017

[B39] Orre M. Kamphuis W. Osborn L. M. Melief J. Kooijman L. Huitinga I. (2014). Acute isolation and transcriptome characterization of cortical astrocytes and microglia from young and aged mice. *Neurobiol. Aging* 35 1–14. 10.1016/j.neurobiolaging.2013.07.008 23954174

[B40] Petravicz J. Fiacco T. A. McCarthy K. D. (2008). Loss of IP3 receptor-dependent Ca2 + increases in hippocampal astrocytes does not affect baseline CA1 pyramidal neuron synaptic activity. *J. Neurosci.* 28 4967–4973. 10.1523/JNEUROSCI.5572-07.2008 18463250 PMC2709811

[B41] Pistell P. J. Spangler E. L. Kelly-Bell B. Miller M. G. de Cabo R. Ingram D. K. (2012). Age-associated learning and memory deficits in two mouse versions of the stone T-maze. *Neurobiol. Aging* 33 2431–2439. 10.1016/j.neurobiolaging.2011.12.004 22217418 PMC3321391

[B42] Rajah M. N. D’Esposito M. (2005). Region-specific changes in prefrontal function with age: a review of PET and fMRI studies on working and episodic memory. *Brain* 128 1964–1983. 10.1093/brain/awh608 16049041

[B43] Rakers C. Petzold G. C. (2017). Astrocytic calcium release mediates peri-infarct depolarizations in a rodent stroke model. *J. Clin. Invest.* 127 511–516. 10.1172/JCI89354 27991861 PMC5272189

[B44] Reichenbach N. Delekate A. Breithausen B. Keppler K. Poll S. Schulte T. (2018). P2Y1 receptor blockade normalizes network dysfunction and cognition in an Alzheimer’s disease model. *J. Exp. Med.* 215 1649–1663. 10.1084/jem.20171487 29724785 PMC5987918

[B45] Rodríguez J. J. Yeh C.-Y. Terzieva S. Olabarria M. Kulijewicz-Nawrot M. Verkhratsky A. (2014). Complex and region-specific changes in astroglial markers in the aging brain. *Neurobiol. Aging* 35 15–23. 10.1016/j.neurobiolaging.2013.07.002 23969179

[B46] Rogers J. T. Liu C.-C. Zhao N. Wang J. Putzke T. Yang L. (2017). Subacute ibuprofen treatment rescues the synaptic and cognitive deficits in advanced-aged mice. *Neurobiol. Aging* 53 112–121. 10.1016/j.neurobiolaging.2017.02.001 28254590 PMC5385269

[B47] Sardinha V. M. Guerra-Gomes S. Caetano I. Tavares G. Martins M. Reis J. S. (2017). Astrocytic signaling supports hippocampal–prefrontal theta synchronization and cognitive function. *Glia* 65 1944–1960. 10.1002/glia.23205 28885722

[B48] Sharp A. H. Nucifora F. C. Blondel O. Sheppard C. A. Zhang C. Snyder S. H. (1999). Differential cellular expression of isoforms of inositol 1,4,5-triphosphate receptors in neurons and glia in brain. *J. Comp. Neurol.* 406 207–220. 10.1002/(SICI)1096-9861(19990405)406:2<207::AID-CNE6>3.0.CO;2-7 10096607

[B49] Soreq L. Rose J. Soreq E. Hardy J. Trabzuni D. Cookson M. R. (2017). Major shifts in glial regional identity are a transcriptional hallmark of human brain aging. *Cell Rep.* 18 557–570. 10.1016/j.celrep.2016.12.011 28076797 PMC5263238

[B50] Srinivasan R. Huang B. S. Venugopal S. Johnston A. D. Chai H. Zeng H. (2015). Ca2 + signaling in astrocytes from Ip3r2-/- mice in brain slices and during startle responses in vivo. *Nat. Neurosci.* 18 708–717. 10.1038/nn.4001 25894291 PMC4429056

[B51] Stobart J. L. Ferrari K. D. Barrett M. J. P. Glück C. Stobart M. J. Zuend M. (2018). Cortical circuit activity evokes rapid astrocyte calcium signals on a similar timescale to neurons. *Neuron* 98 726.e4–735.e4. 10.1016/j.neuron.2018.03.050 29706581

[B52] Takata N. Mishima T. Hisatsune C. Nagai T. Ebisui E. Mikoshiba K. (2011). Astrocyte calcium signaling transforms cholinergic modulation to cortical plasticity in vivo. *J. Neurosci.* 31 18155–18165. 10.1523/JNEUROSCI.5289-11.2011 22159127 PMC6634158

[B53] Tanaka M. Shih P.-Y. Gomi H. Yoshida T. Nakai J. Ando R. (2013). Astrocytic Ca2 + signals are required for the functional integrity of tripartite synapses. *Mol. Brain.* 6:6. 10.1186/1756-6606-6-6 23356992 PMC3563617

[B54] Verkhratsky A. Zorec R. Rodríguez J. J. Parpura V. (2016). Astroglia dynamics in ageing and Alzheimer’s disease. *Curr. Opin. Pharmacol.* 26 74–79. 10.1016/j.neurobiolaging.2016.05.003 26515274

[B55] Volterra A. Liaudet N. Savtchouk I. (2014). Astrocyte Ca2 + signalling: an unexpected complexity. *Nat. Rev. Neurosci.* 15 327–335. 10.1038/nrn3725 24739787

[B56] Wang D. Bordey A. (2008). The astrocyte odyssey. *Prog. Neurobiol.* 86 342–367. 10.1016/j.pneurobio.2008.09.015 18948166 PMC2613184

[B57] Weber M. Wu T. Hanson J. E. Alam N. M. Solanoy H. Ngu H. (2015). Cognitive deficits, changes in synaptic function, and brain pathology in a mouse model of normal aging. *eNeuro* 2: ENEURO.0047-15.2015. 10.1523/ENEURO.0047-15.2015 26473169 PMC4606159

[B58] Wefelmeyer W. Puhl C. J. Burrone J. (2016). Homeostatic plasticity of subcellular neuronal structures: from inputs to outputs. *Trends Neurosci.* 39 656–667. 10.1016/j.tins.2016.08.004 27637565 PMC5236059

[B59] Yu X. Taylor A. M. W. Nagai J. Golshani P. Evans C. J. Coppola G. (2018). Reducing astrocyte calcium signaling in vivo alters striatal microcircuits and causes repetitive behavior. *Neuron* 99 1170.e9–1187.e9. 10.1016/j.neuron.2018.08.015 30174118 PMC6450394

[B60] Zhang Y. Chen K. Sloan S. A. Bennett M. L. Scholze A. R. O’Keeffe S. (2014). An RNA-sequencing transcriptome and splicing database of glia, neurons, and vascular cells of the cerebral cortex. *J. Neurosci.* 34 11929–11947. 10.1523/JNEUROSCI.1860-14.2014 25186741 PMC4152602

